# Sickness Absence Due to Otoaudiological Diagnoses and Risk of Disability Pension: A Nationwide Swedish Prospective Cohort Study

**DOI:** 10.1371/journal.pone.0029966

**Published:** 2012-01-12

**Authors:** Emilie Friberg, Catarina Jansson, Ellenor Mittendorfer-Rutz, Ulf Rosenhall, Kristina Alexanderson

**Affiliations:** 1 Division of Insurance Medicine, Department of Clinical Neuroscience, Karolinska Institutet, Stockholm, Sweden; 2 Department of Clinical Neuroscience, Center for Hearing and Communication Research, Karolinska Institutet, Stockholm, Sweden; 3 Department of Audiology, Karolinska University Hospital, Stockholm, Sweden; Johns Hopkins Bloomberg School of Public Health, United States of America

## Abstract

**Background:**

Hearing difficulties are a large public health problem. Knowledge is scarce regarding risk of disability pension among people who have been sickness absent due to these difficulties.

**Methods:**

A cohort including all 4,687,756 individuals living in Sweden in 2005, aged 20–64, and not on disability or old-age pension, was followed through 2009. Incidence rate ratios (RR) of disability pension with 95% confidence intervals (CI) were estimated using Cox proportional hazard models.

**Results:**

In multivariable models, individuals who had a sick-leave spell due to otoaudiological diagnoses in 2005 had a 1.52-fold (95% CI: 1.43–1.62) increased risk of being granted a disability pension compared to individuals on sick leave due to other diagnoses. Hearing and tinnitus sick-leave diagnoses were associated with risk of disability pension: RR 3.38, 95% CI: 3.04–3.75, and 3.30, 95% CI: 2.95–3.68, respectively. No association was observed between sick leave due to vertigo diagnoses and disability pension whereas otological diagnoses and no sick leave were inversely associated with risk of disability pension compared to non-otoaudiological sick-leave diagnoses. Sick leave due to otoaudiological diagnoses was positively associated with risk of disability pension due to otoaudiological diagnoses and sick leave due to a tinnitus diagnosis was also associated with risk of disability pension due to mental diagnoses. The risk of disability pension among individuals with hearing or tinnitus sick-leave diagnoses was highest in the age group 35–44. Moreover, men had a slightly higher risk.

**Conclusion:**

This large cohort study suggests an increased risk of disability pension among those with sickness absence due to otoaudiological diagnoses, particularly hearing and tinnitus diagnoses, compared to those with sickness absence due to non-otoaudiological diagnoses.

## Introduction

Hearing difficulties are a public health concern [1] with increasing prevalence according to several studies [1], [2], [3], [4], [5]. Other otoaudiological diagnoses, such as vertigo and balance problems, are also important health problems that might cause long-term disability and exclusion from the labor market. In Sweden, approximately half of the persons with hearing difficulties are of working ages [6]. Gender differences have been suggested, i.e. hearing difficulties are more common among men but the prevalence of hearing difficulties is increasing to a larger extent among women [6]. It has been suggested that people with hearing difficulties are overrepresented among those on long-term sick leave [7].

Evidence regarding consequences of being on sick-leave due to different otoaudiological diagnoses is scarce [8]. To our knowledge, there are only two previous studies regarding sickness absence due to hearing difficulties in relation to disability pension [9], [10]. One study examined only vertigo [9] and in the other we found that those who in 1985 had at least one new sick-leave spell due to an otoaudiological diagnosis (mainly vertigo diagnoses) had a higher risk of disability pension compared to individuals on sick leave due to all other diagnoses [10]. In the present, more recent and much larger, nationwide, prospective cohort study we further examine this finding, for the first time adjusting for a large variety of sociodemographic and health variables. The larger number of individuals admits a more specific diagnosis categorization, and, in addition, further possibilities for stratifications. Furthermore, this is the first study examining the association between sick leave due to otoaudiological diagnoses and risk of diagnosis-specific disability pension.

Thus, the objective was to assess if being on sick-leave due to different otoaudiological diagnoses is associated with risk of all-cause disability pension as well as diagnosis-specific disability pension, compared to sickness absence with non-otoaudiological diagnoses, in a Swedish, nationwide, prospective cohort study. Further, the aim was to assess if the potential associations differ with age or sex.

## Methods

All 4 687 756 individuals living in Sweden during 2005 and aged 20–64, and not on disability pension (560 912 individuals) or early old-age pension (29 011 individuals) were included in the cohort. For each individual we had the following annual data: From Statistics Sweden; information on sex, age, educational level, family situation, type of living area, country of birth, migration, and old-age pension. From the Swedish Social Insurance Agency: data on all sick-leave spells compensated in 2005, and disability pension granted in 2006–2009, information includes diagnosis, and old-age pension. Information from the National Patient Register on number of days hospitalized during 2000–2005 and from the national Causes of Death Register on date of death. The unique Personal Identity Number, assigned to all residents in Sweden, was used to link data from the different registers. In Sweden, a sickness certificate issued by a physician is required to obtain sickness benefits after the 7^th^ day of a sick-leave spell. All individuals in Sweden receiving an income from work or from unemployment benefits are covered by the same public sickness insurance. For most employed people, the sickness benefits are compensated by the employer for the first 14 sick-leave days and from the Social Insurance Agency from the 15^th^ day. Disability pension may be granted to those with a medical work incapacity that is predicted to be long term. The old-age retirement age is generally 65 years but may be taken in younger ages. Sickness benefits cover about 80% of lost income and disability and old-age pension at least 65%.

The sick-leave diagnoses were classified according to the International Statistical Classification of Diseases and Related Health Problems, version 10 (ICD-10) [11] (table S1). The exposure was categorized as either: 1) having at least one prevalent (i.e. on-going or new) sick-leave spell during 2005 due to otoaudiological diagnoses (ICD-10 chapter VIII, H60–H95); 2) having at least one prevalent sick-leave spell during 2005 due to non-otoaudiological diagnoses (or no registered diagnosis), reference category), or 3) having no sick-leave day compensated by the Social Insurance Agency during 2005. All individuals with an otoaudiological sick-leave spell were included in the first category, even if they also had non-otoaudiological sick-leave spells during 2005.

Moreover, for detailed analyses, the otoaudiological diagnoses were further categorized as (replacing the category otoaudiological diagnoses in the model): mainly “otological” (H60–H75, H80, H92, H94, H95), “hearing” (H83, H90, H91), “vertigo” (H81, H82), or “tinnitus” (H93). Twenty-seven individuals had more than one sick-leave spell due to two different otoaudiological diagnoses and were thus included in both categories.

The outcome, i.e. disability pension, was defined as incident disability pension, i.e. a disability pension granted during 2006–2009. We studied all-cause disability pension and disability pension due to the following diagnoses: otoaudiological (ICD-10; H60–H95), circulatory (ICD-10;I00-I99), cancer (ICD-10; C00-D48), and mental (ICD-10; F00-F95) diagnoses.

### Statistical analysis

We calculated person-time of follow-up for each individual from January 1, 2006 to the date of disability pension, death, migration, 65 years of age, or December 31, 2009, whichever came first. Incidence rate ratios (RRs) for all cause and diagnosis-specific disability pension and 95% confidence intervals (CIs) were estimated by Cox proportional hazards models, using person-time of follow-up as the underlying time scale. The data conformed to the proportional hazards assumption.

We performed crude, age-adjusted, and multivariable analyses. In the main analysis, we adjusted for age, sex, family situation, type of living area, country of birth, educational level, and number of days hospitalized (inpatient) in 2000–2005. Furthermore, the risk of diagnosis-specific disability pension associated with sick leave due to different otoaudiological diagnoses was assessed. Moreover, analyses stratified by age and sex was performed for all-cause disability pension since both age and sex have been reported to influence the risk of both sick-leave and disability pension [12]. All individuals with missing information on any of the variables included in the models were excluded from the analyses.

All statistical analyses were performed using SAS software (version 9.2; SAS Institute, Cary, NC).

The study was approved by the Regional Ethical Committee in Stockholm, Sweden.

## Results

**Figure 1:**
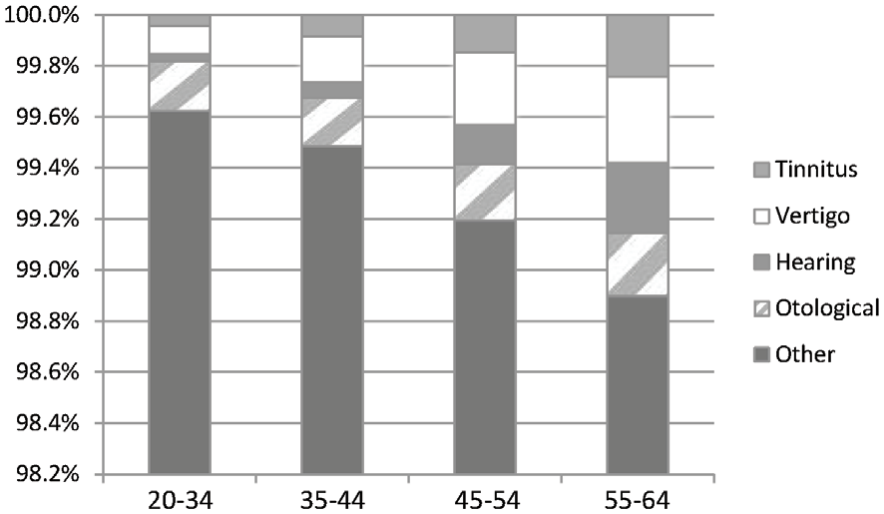
Distribution (%) of individuals sickness absent due to different sick-leave diagnoses in four different age groups. Otological: (ICD10) H60–H75, H80, H92, H94, H95. Hearing: (ICD10) H83, H90, H91. Vertigo: (ICD10) H81, H82. Tinnitus: (ICD10) H93. Other: all non-otoaudiological sick-leave diagnoses.

The 4 687 756 individuals together contributed 17 918 476 person-years during the mean follow-up of 3.8 years (2006–2009). In total, 123 473 individuals were granted disability pension of which 1709 individuals were granted disability pension due to otoaudiological diagnoses. The mean age when granted disability pension (due to all diagnoses) was 50 (±11) and due to an otoaudiological diagnosis 55 (±8). In table 1, baseline characteristics of the cohort, in total and by the different categories of sick-leave diagnoses, are presented. Individuals with at least one sick-leave spell during 2005 due to an otoaudiological diagnosis were on average older, more often married/cohabiting, born in another country than Sweden, less often living in a large city or hospitalized compared to individuals with a sick-leave spell with non-otoaudiological diagnoses in 2005. Individuals with a hearing or tinnitus sick-leave diagnosis had on average a higher educational level compared to both other sickness absentees and to those with no sickness absence benefits in 2005. Among those with sickness absence in 2005, the proportion absent due to otoaudiological diagnoses increased with age (figure 1).

**Table 1:** Baseline characteristics for all individuals (n = 4 687 756) included in the cohort in 2005.

Characteristic	All	No sick leave in 2005^a^	Sick-leave due to non-otoaudiological diagnoses^a^	Sick-leave due to otoaudiological diagnoses^a^ ^b^				
				All	Otological^c^	Hearing^d^	Vertigo^e^	Tinnitus^f^
N	4 687 756	4 078 640	604 951	4 165	1 283	770	1 372	767
(%)	(100)	(87.01)	(12.90)	(0.009)	(0.0002)	(0.0002)	(0.0003)	(0.0002)
Women (%)	48	46	61	58	59	63	61	47
Mean age (years)	41	41	44	48	45	52	48	50
Education >12years (%)	36	37	27	32	26	42	31	41
Living in larger cities (%)	38	38	35	35	38	29	36	31
Not born in Sweden (%)	14	14	15	14	18	12	13	10
Married or cohabiting (%)	56	56	58	57	61	56	64	58
Days hospitalized in 2000–2005	1.85	1.30	5.61	3.41	3.94	3.41	3.66	1.95

a Values other than for age and sex have been directly standardized according to the age distribution of the cohort.

b Twenty-seven individuals had more than one spell of sick leave and two different otoaudiological diagnoses and thus are included in two categories.

c ICD10: H60–H75, H80, H92, H94, H95.

d ICD10: H83, H90, H91.

e ICD10: H81, H82.

f ICD10: H93.

Overall, we observed that individuals with sick-leave due to any otoaudiological diagnosis had an increased risk of being granted a disability pension (RR 1.52, 95% CI 1.43–1.62) compared to individuals with sick-leave due to non-otoaudiological diagnoses (table 2). Especially sickness absence due to hearing and tinnitus diagnoses were associated with high risk increases of disability pension; RR 3.38 (95% CI 3.04–3.75) and 3.30 (95% CI 2.95–3.68), respectively. Sick leave due to vertigo diagnoses was associated with modest risk increases in the crude models, but not in the multivariable adjusted models and sickness absence due to otological diagnoses was inversely associated with risk of disability pension compared to sickness absence due to non-otoaudiological diagnoses. Inverse associations were observed between no sick-leave days and risk of disability pension compared to those with non-otoaudiological sick leave.

**Table 2:** Sickness absence due to otoaudiological diagnoses and incidence rate ratios (RRs) and 95% confidence intervals (CIs) for disability pension.

	Number of people granted disability pension	Person-years	Crude RR (95% CI)	Age-adjusted RR (95% CI)^a^	Multivariable RR (95% CI)^b^	Multivariable RR (95% CI)^c^
Non-otoaudiological sick-leave diagnoses	89 044	2 121 525	1.00 (ref)	1.00 (ref)	1.00 (ref)	1.00 (ref)
No sick leave	33 435	15 783 423	0.05 (0.05–0.05)	0.06 (0.06–0.06)	0.06 (0.06–0.06)	0.07 (0.07–0.07)
All otoaudiological sick-leave diagnoses	994	13 528	1.73 (1.62–1.84)	1.45 (1.36–1.54)	1.52 (1.43–1.62)	1.52 (1.43–1.62)
Otological^d^	90	4 799	0.44 (0.36–0.55)	0.41 (0.34–0.51)	0.42 (0.34–0.52)	0.40 (0.33–0.49)
Hearing^e^	352	1 979	3.93 (3.54–4.36)	2.90 (2.61–3.22)	3.20 (2.88–3.56)	3.38 (3.04–3.75)
Vertigo^f^	249	4 694	1.24 (1.09–1.40)	1.04 (0.92–1.18)	1.06 (0.93–1.20)	1.03 (0.91–1.16)
Tinnitus^g^	318	2 126	3.34 (2.99–3.73)	2.61 (2.34–2.91)	2.97 (2.65–3.31)	3.30 (2.95–3.68)

a Adjusted for age (20–34, 35–44, 45–54, 55–64).

b Further adjusted for sex (female, male); family situation (married/cohabiting, married/cohabiting with children, single, single with child, child living with parent); type of living area (larger cities, medium sized cities, smaller places); birth region (Sweden, Nordic countries, Europe, Other world), years of education (0–9, 10–12, 12<), excluding 292 403 observations due to missing data on education and/or family situation.

c Further adjusted hospitalization days (summarized days 2000–2005; 0, 1–4, >4), excluding 292 403 observations due to missing data on education and/or family.

d ICD10: H60–H75, H80, H92, H94, H95.

e ICD10: H83, H90, H91.

f ICD10: H81, H82.

g ICD10: H93.

In the analyses of diagnosis-specific disability pension (table 3), we observed strongly increased risks of incident disability pension due to otoaudiological diagnoses among those sickness absent due to these diagnoses. Although the inverse association between no sick leave benefits and risk of disability pension due to otoaudiological diagnoses compared to sickness absence due to non-otoaudiological diagnoses were strong, it was weaker than in the analyses of disability pension due to other outcomes. A sick-leave diagnosis of tinnitus was associated with an increased risk of disability pension due to mental diagnoses (RR 1.69, 95% CI 1.27–2.25), while the other otoaudiological diagnoses categories were not. Very few individuals with a sick-leave spell due to otoaudiological diagnoses were granted a disability pension due to circulatory (20 individuals) or cancer (10 individuals) diagnoses. A sick-leave spell due to otoaudiological diagnoses was associated with decreased risks of disability pension due to circulatory (RR 0.42, 95% CI 0.27–0.65) and cancer (RR 0.43, 95% CI 0.23–0.79) diagnoses.

**Table 3:** Sickness absence due to otoaudiological diagnosis and incidence rate ratios (RR) with 95% confidence intervals (CI) for diagnosis specific disability pension in 2006–2009 among individuals living in Sweden.

	Number of people granted diagnosis-specific disability pensions	Crude RR (95% CI)	Multivariable RR (95% CI)^a^
**Disability pension due to otoaudiological diagnoses**	(1709)		
All non-otoaudiological sick-leave diagnoses	509	1.00 (ref)	1.00 (ref)
No sick leave	523	0.14 (0.12–0.16)	0.15 (0.13–0.17)
All otoaudiological sick-leave diagnoses	677	205.72 (183.37–230.79)	147.76 (131.45–166.10)
Otological^b^	35	12.01 (8.57–16.82)	11.08 (7.90–15.54)
Hearing^c^	288	168.29 (146.01–193.98)	107.56 (93.16–124.18)
Vertigo^d^	138	51.06 (42.66–61.10)	43.24 (36.14–51.73)
Tinnitus^e^	229	118.41 (101.47–138.19)	74.24 (63.57–86.69)
**Disability pension due to mental diagnoses**	(45 234)		
All non-otoaudiological sick-leave diagnoses	31 182	1.00 (ref)	1.00 (ref)
No sick leave	13 933	0.06 (0.06–0.06)	0.07 (0.07–0.07)
All otoaudiological sick-leave diagnoses	119	0.59 (0.49–0.71)	0.58 (0.48–0.69)
Otological^b^	14	0.20 (0.12–0.34)	0.19 (0.11–0.32)
Hearing^c^	24	0.78 (0.52–1.16)	0.78 (0.52–1.16)
Vertigo^d^	33	0.48 (0.34–0.67)	0.45 (0.32–0.64)
Tinnitus^e^	48	1.48 (1.12–1.97)	1.69 (1.27–2.25)

a Adjusted for age (20–34, 35–44, 45–54, 55–64); sex (female, male); family (married/cohabiting, married/cohabiting with children, alone, alone with child, child living with parent); type of living area (larger cities, medium sized cities, smaller places); birth region (Sweden, Nordic countries, Europe, Other world); years of education (0–9, 10–12, >12 ); hospitalization days (summarized days 2000–2005; 0, 1–4, >4), excluding 292 403 observations due to missing data on education and/or family.

b ICD10: H60–H75, H80, H92, H94, H95.

c ICD10: H83, H90, H91.

d ICD10: H81, H82.

e ICD10: H93.

Finally, in the analyses of all disability pension stratified by age and sex, respectively, we observed relatively small differences (table 4). The risk of being granted a disability pension among individuals with a hearing or tinnitus diagnosis was highest in the age-group 35–44. Men who were sickness absent due to otoaudiological diagnoses had a slightly higher risk of disability pension than women sickness absent with these diagnoses.

**Table 4:** Multivariable incidence rate ratios^a^ with 95% confidence intervals for disability pension in 2006–2009 among individuals with sickness absence due to otoaudiological diagnosis in 2005, stratified by age and sex.

	N	Non-otoaudiological sick-leave diagnoses	No sick leave	Otoaudiological sick-leave diagnoses				
				All	Otological^b^	Hearing^c^	Vertigo^d^	Tinnitus^e^
Age								
20–34	1 368 888	1.00 (ref)	0.07	1.52	0.31	1.17	0.71	2.21
			(0.07–0.08)	(1.43–1.62)	(0.14–0.69)	(0.38–3.62)	(0.34–1.49)	(0.99–4.93)
35–44	1 153 491	1.00 (ref)	0.05	1.17	0.37	3.70	0.75	3.48
			(0.05–0.06)	(0.97–1.42)	(0.22–0.62)	(2.66–5.15)	(0.51–1.11)	(2.50–4.85)
45–54	995 615	1.00 (ref)	0.06	1.46	0.41	3.19	1.15	3.19
			(0.06–0.06)	(1.30–1.64)	(0.28–0.60)	(2.61–3.89)	(0.93–1.41)	(2.58–3.95)
55–64	877 059	1.00 (ref)	0.07	1.70	0.42	3.34	1.04	3.17
			(0.07–0.07)	(1.57–1.85)	(0.31–0.56)	(2.92–3.83)	(0.87–1.24)	(2.75–3.66)
Sex								
Women	2 146 123	1.00 (ref)	0.06	1.48	0.45	3.23	1.01	3.17
			(0.06–0.07)	(1.36–1.60)	(0.35–0.58)	(3.84–4.67)	(0.86–1.18)	(2.70–3.71)
Men	2 248 930	1.00 (ref)	0.07	1.60	0.32	3.80	1.06	3.50
			(0.07–0.07)	(1.45–1.77)	(0.22–0.46)	(3.16–4.56)	(0.86–1.29)	(3.00–4.09)

a Adjusted for age (20–34, 35–44, 45–54, 55–64) or sex (female, male) when applicable, and family (married/cohabiting, married/cohabiting with children, alone, alone with child, child living with parent); type of living area (larger cities, medium sized cities, smaller places); birth region (Sweden, Nordic countries, Europe, Other world); years of education (0–9, 10–12, >12); hospitalization days (summarized days 2000–2005; 0, 1–4, >4).

b ICD10: H60–H75, H80, H92, H94, H95.

c ICD10: H83, H90, H91.

d ICD10: H81, H82.

e ICD10: H93.

## Discussion

In this population-based cohort including all individuals living in Sweden, aged 20–64 years, and not on old-age or disability pension in 2005, we found that the risk of being granted a new disability pension was 52% higher among individuals having at least one sick-leave spell due to an otoaudiological diagnosis compared to individuals with sickness absence due to non-otoaudiological diagnoses. The risk increase was most pronounced among individuals with a hearing or tinnitus diagnosis, where more than threefold increased risks of being granted a disability pension compared to those with non-otoaudiological sick-leave diagnoses were observed. As expected, the risk of diagnosis-specific disability pension due to otoaudiological diagnoses was very strongly associated with sick-leave due to otoaudiological diagnoses. The associations did not differ substantially across different age groups or between men and women.

Only a few prior cohort studies have presented data on sick leave or disability pension in relation to hearing difficulties or diagnoses [9], [10], [13], [14], [15], [16], [17]. The majority of previous studies include small groups [9], [13], [14], [16] or only focus on specific occupational groups [15], [17]. Most previous studies [9], [10], [15], [16] have indicated an increased risk of disability pension among individuals with a hearing difficulty. The results of this study confirms and extends previous results from a Swedish cohort study including 40 786 individuals living in a county in southern Sweden, where we found that individuals with sick leave due to an otoaudiological diagnosis had a 42% higher risk of disability pension compared to individuals with sick leave spells due to non-otoaudiological diagnoses [10].

One explanation for our findings is that hearing difficulties lead to increased levels of stress. Even a minor hearing difficulty might decrease an individual's possibilities to be involved in ordinary conversation, especially if there is several persons talking at the same time or if there is a lot of background noise, the need to constantly strain to hear might be stressful [6].

As in all studies on sick leave due to specific diagnoses the number of persons on sick leave with a specific diagnosis cannot directly be translated to all who have that disease. Most people with different diseases, including hearing difficulties, are not sickness absent due to them [18], [19]. Most likely, a majority of people with hearing difficulties have different aids or adapted work conditions which means that they may be able to work regardless of their hearing difficulties or their hearing difficulties does not impair their work capacity. Moreover, for some individuals with multi-morbidity, hearing difficulties might not have been the main reason for their sick leave, but contributed to their work incapacity. This means that our results might be an underestimation of the associations observed between being on sick-leave due to otoaudiological diagnoses and risk of disability pension.

Major strengths of this study include its population-based nationwide design, comprising the whole Swedish population of working ages, not a sample, the prospective study design, and the complete follow-up (i.e. no drop out). Other strengths are the high quality of the population-based registers, that data were not self-reported [20], [21], and our adjustments for a wide range of potential confounders such as age, sex, family situation, type of living area, birth region, years of education and, hospitalization days. The prospective design and the register-based information including the whole Swedish population limit the risk of selection bias. Another strength is that detailed information regarding sick-leave diagnoses was available. Such information is often not available in large population-based studies. The validity of sick-leave diagnoses is often discussed, however, seldom studied. A study of the validity of sick-leave diagnoses comparing them to medical charts report good validity [22].

A limitation is that for employed individuals, information about most sick-leave spells shorter than 15 days are not included. However, the majority of shorter sick-leave spells are due to different types of infections, migraine etc. [12]. If such sick-leave spells had been included, the associations would probably have been even stronger. Another limitation is that we excluded persons who already had received or were granted disability pension during 2005. This might have underestimated the risk of being granted a disability pension. The relatively short follow-up of four years has advantages and disadvantages, but a 52% risk increase of all cause disability pension among those on sick leave due to otoaudiological diagnoses warrants attention. The register-based data does not include lifestyle factors that might influence the risk of being granted a disability pension, thus the possibility of uncontrolled or residual confounding cannot be entirely eliminated. However, we have adjusted for multiple potential confounders, and we observed only very small differences in the risk estimates between the crude models and the adjusted models.

In absolute numbers, the number of individuals with sick-leave spells due to audiological diagnoses (i.e. hearing loss or tinnitus) might not be considered very large, but the risk of exclusion from the labor market and the adverse consequences must be recognized. In our study, many individuals aged 35–40 years with such sick-leave diagnoses were granted disability pension. This observation is consistent with the notion that many of the subjects could have early onset hearing loss that may progress into profound hearing loss, or profound hearing loss with adult onset [23]. The severe consequences of a disrupted ability to communicate due to late deafness are well known [24]. Awareness of the importance to introduce work adjustments or comprehensive rehabilitation measures is warranted.

In conclusion, this large nationwide population-based cohort study suggests an increased risk of disability pension among those sickness absent due to otoaudiological diagnoses compared to those sickness absent due to non-otoaudiological diagnoses. The risk increase was most pronounced among individuals with a hearing or a tinnitus sick-leave diagnosis. This finding emphasizes the importance of initiating preventive measures among people with sick leave due to otoaudiological diagnoses in order to prevent disability pension.

## Supporting Information

Table S1 **Categorization of otoaudiological diagnoses (Chapter VIII. Diseases of the ear and mastoid process) in accordance with the International Statistical Classification of Diseases and Related Health Problems 10th Revision (ICD-10).**
(DOCX)
