# Supplementary material for: Sickness Absence Due to Otoaudiological Diagnoses and Risk of Disability Pension: A Nationwide Swedish Prospective Cohort Study
Source: PLoS One. 2012 Jan 12;7(1):e29966. doi: 10.1371/journal.pone.0029966 (PMC3257229; doi:10.1371/journal.pone.0029966)
Supplement: Table S1 — Categorization of otoaudiological diagnoses (Chapter VIII. Diseases of the ear and mastoid process) in accordance with the International Statistical Classification of Diseases and Related Health Problems 10th Revision (ICD-10). (DOCX) [file pone.0029966.s001.docx]

**Table S1.**

Categorization of otoaudiological diagnoses (Chapter VIII. Diseases of the ear and mastoid process) in accordance with the International Statistical Classification of Diseases and Related Health Problems 10th Revision (ICD-10).

| Otological: (ICD-10) | H60 Otitis externa |
| --- | --- |
|  | H61 Other disorders of external ear |
|  | H62 Disorders of external ear in diseases classified elsewhere |
|  | H65 Nonsuppurative otitis media |
|  | H66 Suppurative and unspecified otitis media |
|  | H67 Otitis media in diseases classified elsewhere |
|  | H68 Eustachian salpingitis and obstruction |
|  | H69 Other disorders of eustachian tube |
|  | H70 Mastoiditis and related conditions |
|  | H71 Cholesteatoma of middle ear |
|  | H72 Perforation of tympanic membrane |
|  | H73 Other disorders of tympanic membrane |
|  | H74 Other disorders of middle ear and mastoid |
|  | H75 Other disorders of middle ear and mastoid in diseases classified elsewhere |
|  | H80 Otosclerosis |
|  | H92 Otalgia and effusion of ear classified elsewhere |
|  | H94 Other disorders of ear in diseases |
|  | H95 Postprocedural disorders of ear and mastoid process, not elsewhere classified |
| Hearing: (ICD-10) | H83 Other diseases of inner ear |
|  | H90 Conductive and sensorineural hearing loss |
|  | H91 Other hearing loss |
| Vertigo: (ICD-10) | H81 Disorders of vestibular function |
|  | H82 Vertiginous syndromes in diseases classified elsewhere |
| Tinnitus: (ICD-10) | H93 Other disorders of ear, not elsewhere classified (including tinnitus and other abnormal auditory perceptions) |
